# Working at the nexus between public health policy, practice and research. Dynamics of knowledge sharing in the Netherlands

**DOI:** 10.1186/1478-4505-10-33

**Published:** 2012-10-17

**Authors:** Maria W Jansen, Evelyne De Leeuw, Marjan Hoeijmakers, Nanne K De Vries

**Affiliations:** 1 Academic Collaborative Centre for Public Health Limburg, Regional Public Health Service, PO Box 2022, Geleen, HA 6160, The Netherlands; 2 School of Public Health and Primary Care: CAPHRI, Maastricht University, Maastricht, The Netherlands; 3 Faculty of Health, School of Medicine, Deakin University, Geelong, Australia; 4 Faculty of Health, Medicine and Life Sciences, Department of Health Education and Promotion, Maastricht University, Maastricht, The Netherlands

**Keywords:** Knowledge sharing, Practice- and policy-based evidence, Evidence-based public health

## Abstract

**Background:**

Joining the domains of practice, research and policy is an important aspect of boosting the quality performance required to tackle complex public health problems. “Joining domains” implies a departure from the linear and technocratic knowledge-translation approach. Integrating the practice, research and policy triangle means knowing its elements, appreciating the barriers, identifying possible cooperation strategies and studying strategy effectiveness under specified conditions.

This article examines the dynamic process of developing an Academic Collaborative Centre for Public Health in the Netherlands, with the objective of achieving that the three domains of policy, practice and research become working partners on an equal footing.

**Method:**

An interpretative hermeneutic approach was used to interpret the phenomenon of collaboration at the nexus between the three domains. The project was explicitly grounded in current organizational culture and routines, applied to nexus action. In the process of examination, we used both quantitative (e.g. records) and qualitative data (e.g., interviews and observations). The data were interpreted using the Actor-Network, Institutional Re-Design and Blurring the Boundaries theories.

**Results:**

Results show commitment at strategic level. At the tactical level, however, managers were inclined to prioritize daily routine, while the policy domain remained absent. At the operational level, practitioners learned to do PhD research in real-life practice and researchers became acquainted with problems of practice and policy, resulting in new research initiatives.

**Conclusion:**

We conclude that working at the nexus is an ongoing process of formation and reformation. Strategies based on Institutional Re-Design theories in particular might help to more actively stimulate managers’ involvement to establish mutually supportive networks.

## Introduction

Since 1989, municipal governments in the Netherlands have been responsible for local public health policy [1]. The implementation of local policy is supported by regional Public Health Services (PHSs). There are 421 municipalities and 28 PHSs in the Netherlands, distributed throughout the entire country (41,848 km^2^, approximate population 16,000,000). The Dutch Health Care Inspectorate has been reporting on health promotion and disease prevention performance at the local level since 1995, but the results have consistently been disappointing [2,3]. For instance, the prevalence of excessive alcohol consumption, i.e. binge drinking, among Dutch youngsters is 35.6%, and smoking rates are 22% for youngsters and 28% for adults, with more women smoking than in other European countries [4,5]. Recent trends in the Netherlands show that between 1980 and 2010, the prevalence of overweight among Dutch girls aged 4-15 years increased from 7.2% to 14.9%, and in boys of the same ages from 5.1% to 13.3%, with the highest prevalence of overweight being observed in boys of Turkish origin (32.5%) [6]. These health problems are associated with chronic diseases such as obesity, type 2 diabetes, cardiovascular diseases, several forms of cancer and asthma. Furthermore, the numbers of healthy life years of people with limited education and low incomes are lagging behind [4]. Local authorities are failing in their efforts to translate the national priorities into effective measures. According to the Inspectorate, local authorities have failed to specify a targeted and systematic approach, and policies have not been evidence-based.

In view of the increasing pressure to use evidence-based public health promotion to counteract the current trends in chronic diseases and health care spending, the Ministry of Health, Welfare and Sport has initiated a programme to support local governments and their PHSs by establishing Academic Collaborative Centres for Public Health [7-9]. Academic Collaborative Centres (ACC) are charged with building a regional, sustainable knowledge-sharing network involving three different institutional partners: (i) local government, which designs health policy and decides on evidence-based service supply and resource allocation; (ii) PHSs, which initiate evidence-based community health promotion and social action projects, and organize service delivery; and (iii) research institutes, which evaluate processes and assess the effectiveness and cost-benefit ratios of practice-based interventions or policy-based measures [8]. A need for more scientific rigour is recognized in the maxim: “if we want more evidence-based practice, we need more practice-based evidence” [10].

Joining the domains of practice, research and policy requires a transformation of patterns of interaction, interpreted as complex responses of humans in their relations with one another. The ACC Limburg was established in 2006, involving nineteen municipalities in the province of Limburg, as well as the regional PHS for Southern Limburg, and the Maastricht University Medical Centre (MUMC+). The aim of this paper is to examine the dynamics of knowledge sharing between the actors involved in the ACC Limburg, as well as the collaboration at the nexus between the three domains, and to suggest improvements.

## Background

Integrating the research, policy and practice domains requires (1) knowing the characteristics of each domain, (2) regarding the differences as a challenge, (3) identifying possible strategies for cooperation and (4) studying their effectiveness under specified conditions.

In a 2006 study, we traced the main characteristics of the policy, practice and research domains. The study revealed a number of challenges encountered by stakeholders at the nexus of these local public health domains. They proved to have diverging ideas about the social, practical or scientifically relevant nature of the problems they were facing; they sometimes had different agendas and priorities; they formulated issues in different ways and set goals according to different standards; they sometimes disagreed on the importance of evidence and research quality parameters (such as validity and reliability); and they had incompatible ideas about the readability and audiences of publications [11,12]. In view of these differences, studies have been conducted to establish commonalities and differences between policy, practice and research, importantly regarding the factors that contribute to what is regarded as successful collaboration in each niche [11,13]. On the basis of this, the ACC Limburg 1) developed strategies to stimulate collaboration between policy, practice and research, and 2) systematically organized practical strategies to establish enduring links between researchers, policy officers and practitioners. These practical strategies could facilitate interactions along the lines of the existing organizational hierarchy, distinguishing the actors at the strategic, tactical and operational levels of each domain [13,14]. The main actors at strategic, i.e. administrative level were councillors, members of the board of the practice institute(s) and the university board of governors. Actors at tactical level were the managers of the policy, practice and research domain. At the operational level the actors were civil servants, practitioners and researchers. The network was intended to operate horizontally on a functional basis, but could not do without the participating organizations, which operate vertically. As the more formal, vertical relations within policy, practice and research organizations already existed as regular (authoritative) decision structures, the more informal, horizontal networks still needed to be established. Twenty-three practical strategies were planned (see table under ‘Results’ section) based on previous research [13]. The project as a whole was intended to shape a social, adaptive and self-organizing approach in which the organization adapts to the collaborative and the collaborative adapts to the organization [15,16]. This requires a socially and organically emerging approach involving knowledge sharing through formal and informal meetings, masterclasses, multidisciplinary working groups and a feeling of ACC-family membership, rather than the “traditional”, perhaps technocratic, knowledge-translation approach [17-25]. Knowledge-translation theories follow a bilateral-stages heuristic, which means that a stepwise procedure between stakeholders supposedly ensures that the existing knowledge is applied in policy and practice, but what is lacking in these is reciprocity, mutual engagement and domain integration.

**Table 1:** Matrix indicating the actors, targets, planned and realized practical strategies during the collaboration process and their match with theories, May 2006-December 2010

**System level**	**Targets**	**Planned practical strategies to reach targets**	**Realized practical strategies**	**Match with basic principles of Actor Network Theory (ANT), theories of Institutional Re-Design (IRD) and Blurring Boundaries (BB) with specification**
**Strategic level**	· Commitment	1. Steering committee and daily board, 4/yr, discussing progress and evaluating strategies	Number of meetings of Steering Committee respectively Daily Board of Collaborative:	ANT - creation of a new platform to communicate; stakeholders at strategic level in dialogue at the moment of problematization
	· Joint decision making		2006 5 resp. 4 meetings, attendance 100%	
**Actors from policy:** councillors, mayor, aldermen	· Create public attention		2007 2 resp. 7 meetings, attendance 90%	
			2008 2 resp. 8 meetings, attendance 80%	BB - a negotiated zone or ‘white space’at strategic level, to develop a climate of trust
			2009 2 resp. 4 meetings, attendance 90%	
			2010 1 (1 cancelled due to extreme wheather) resp. 5 meetings, attendance 80%.	
**Actors from research:** university board orprofessor ordirector of research institute			Lobbying efforts in 24 out of 34 min. Decisions were shared among all actors.	
		2. Contractual agreement	Signed by faculty governors, board of governors of PHS and local governments	IRD - decision on changing the rules of the game in global terms
		3. Kick-off meeting, at start	March 21^th^, 2007 (n=65 participants from the three domains, mainly from strategic and tactical levels). Spirit of innovation. Aldermen stressed focus on the lot of the downtrodden.	ANT - adding an new communication platform at the moment of problematization
**Actors from practice:** Public Health Service board and managing director		4. Symposium half way	May 28^th^, 2009 (n= 115 participants from the three domains, from all levels). Spirit of active learning. Continuation of investments, promised by the dean of the faculty.	ANT - adding a new communication platform to inspire each other by crossing domain boundaries at the moment of problematization a
		5. Ambassadors, 1 per project	In 2007 seven ambassadors. After municipal council elections in 2010 two ambassadors stayed in function.	IRD - giving the alderman a new task within the research domain
		6. Content meetings between professors and alderman, 2-3/yr	Totally 10 meetings on topics like alcohol prevention, youth health problems, mental public health, population health forecast, preventive care for elderly, cardiovasculair disease prevention, the future of public health, health statistics, health in all policies, overweight prevention	ANT - creation of a new platform to communicate about complex public health problems
		7. Newsletter, 10/yr	Totally 35 newsletters (n=500) members from the three domains, at all levels	ANT - adding a new communication channel to inform each other about activities at the nexus at the moment of problematization
		8. Publications in local media and professional media, 2-8/yr	Totally 201 publications about research topics in practice or policy. Plus a book about four years experience within the Limburg collaborative. Local officials felt honored to be presented in the book.	ANT - regularly send out messages to inform partners about working at the nexus, also to maintain commitment at strategic level (PR-instrument)
		9. Oral presentations at local and national podia about the collaborative, 2-3/yr	Totally 24 presentations. One presentation as keynote speaker at the national public health congress by the alderman of the city Maastricht.	IRD - new tasks for strategic, tactical and operational actors from policy and practice to present at research conferences, also to maintain commitment at strategic level (PR-instrument)
		10. Interface meetings on content, 2-3/yr	Started in 2009, totally 6 (mean n=75 participants from the three domains, from all levels). Joint discussion on research findings related to questions based on real-life situations and local context.	IRD - new tasks for tactical and operational actors from policy, practice and research to present research results at interface meetings and to discuss these results from the perspective of policy, practice and research
		11. Website	http://www.academischewerkplaatslimburg.nl	ANT - adding a new communication channel to the collaborative
**Tactical level**	· Reset culture of policy and practice into lifelong learning culture	12. Annual plans jointly made by 4 thematic groups led by PHS and university managers	Started in 2009, totally 3 annual plans in 2009. Quality: annual plan on Infectious Disease according standard quality, annual plans on Health Promotion/Local Policy respectively Youth Healh Care below standard quality. No annual plan for Preventive Care for the Elderly. No annual plans in 2010.	IRD - a new rule for each organization to make a joint annual plan
**Actors from policy:** managers of civil service departments	· Organize prerequisites for collaboration, mutual service delivery and (in)formal consultations between municipalities, PHS and university			
	· Use role models to share values and beliefs			
**Actors from research:** professors as managers of research department		13. Preparation and submission of new practice-based research proposals	Totally 37 proposals submitted of which 29 were granted with a total budget of 8 million Euro (range 25.000 to 2.000.000 Euro).	IRD - a new rule for each organization to prepare and submit research proposals based on local context and policy or practice-based problems in public health
		14. Dual-staff appointments for PHS-professionals	Totally 17 PHS-professionals have a dual-staff or honorary appointement at Maastricht University, giving them entrance to university library, research soft ware applications, training modules and expertise of university staff. Academics were given entrance to data.	IRD - a new facility for practitioners and policymakers to get entrance to the research domain
**Actors from practice:** Public Health Service managers			All masterclass students (n=21) had a dual-staff appointment during the period Jan. 2008 - Jan. 2010	ANT - a new linkage between actors from practice and research
				IRD - a new facility for academics to use public health data sources
				BB - to facilitate entrance to each others domain
		15. Flexible workstations for PHS and university according needs	Totally 8 flexible workstations realized, availability according the needs	IRD - a new facility for practitioners and policymakers to become actively enrolled in the research domain
				BB - to facilitate entrance to each others domain and culture
		16. Role models	Three professionals from practice were awarded. Candidates for the Spreeuw Award (the best practice-based research), the Crebolder Award (research ranking highest in societal impact) and the Philipsen Award (the best article). Next, 16 masterclass students and 16 PhD-trainees function as a role model in newsletters and website.	ANT - communication about a new culture of lifelong learning and working beyond routine
		17. Agendasetting in regular meetings with municipality staff, initiated by PHS staff, 4x/yr	Totally 33 topics in 14 meetings. Mainly informative, without deep discussion. Intensified collaboration between 3 municipalities to work out research proposals. One of the three proposal was accepted.	IRD - introduction of research issues in regular meetings between policy and practice domain
		18. Facilitation of participation in masterclasses or PhD-training to upgrade competence of PHS professionals	Totally 16 particpants in practice-based research masterclass of whom 13 were supported by their employer. Totally 16 researchers in PhD training of whom 10 are facilitated by their employer to work in practice and combine regular work with PhD-research with job garantee. Six persons have a timely research position for 4 yrs.	IRD - a new facility to train professionals on the job
**Operational level Actors from policy:** civil servants	· Active learning and knowledge sharing	19. Participation in masterclasses or PhD training	Totally 16 particpants in practice-based research masterclass. Next, 16 researchers in PhD training of whom 10 spent 50% of time on research and 50% on regular work. Six researchers fully work at the university as PhD candidate, doing research in practice.	ANT - a new linkage between actors from practice and research with resources and cultures from other domains
	· Sharing of capabilties/competences			
	· Broadening personal network			BB - giving access to the internal structure and culture of each other domain, mitigation of inequalities among partners, a negotiated zone to develop a climate of trust, active enrolment in each other domain


**Actors from research:** researchers		20. Multidisciplinary working groups around each PhD candidate, combining expertise from policy, practice and research	Totally 16 working groups started of which 8 combined professionals from the domains of policy, research and practice; 2 continued as such after 2010. The other 8 combined the domains of research and practice, 4 continued as such after 2010.	BB - giving acces to the internal structure and culture of each other domain, mitigation of inequalities among partners, a negotiated zone at operational and tactical level to develop a climate of trust
**Actors from practice:** practitioners		21. Oral presentations about research topics at national and international congresses 5/yr	Totally 117 oral presentations with published abstracts of practice-based research (international n=67, national n=50)	IRD - new tasks for practitioners and policymakers to present at research conferences
		22. Peer-reviewed publications in (inter) national journals, 5/yr	Totally 73 peer-reviewed publications, majority focused on infectious disease, minority on school and community health promotion: 51 in international and 22 in national journals	IRD - new tasks for practitioners and policymakers to publish in peer-reviewed international journals
**Switching between all levels**	· Social entrepreneurship	23. Program leader	Appointment of program leader at full time base, paid by municipalities (80%) and university (20%)	BB - a mediator to develop a climate of trust and to support other partners in a process which requires considerable efforts over a rather long period of time

To study the strengths and weaknesses of our knowledge-sharing approach, all strategies were assessed from the perspective of three major theories about working at the nexus of policy, practice and research, viz. the Actor-Network Theory (ANT), theories about Institutional Re-Design (IRD) and Blurring the Boundaries (BB) [26].

ANT offers a comprehensive framework for the analysis of fundamental system change. To change systems, actors and resources must be transformed away from their formal subsystem roles [27]. ANT stresses the importance of creating a network of actors and resources that grows as they join with others, outside the system, to pursue the innovation. ANT fits in with the social, adaptive and self-organizing approach we apply for the ACC since from an ANT perspective, none of the elements of the ACC is fixed beforehand. ANT explains how the ACC elements come together to act as a whole after having passed a mandatory point of entry where everyone is forced to trade, i.e. the “obligatory passage point”. Actor networks are potentially transient, existing in a constant process of making and re-making [27,28]. To analyze the dynamics within the ACC, we restricted ourselves to the ANT notion of problematization, which is only the first of four successive steps, viz. problematization, interest, enrolment and mobilization of allies. Problematization means that the problem is constructed in a reiterative process in which actors can always (implicitly or explicitly) ignore the problem or present it in a different way.

The Institutional Re-Design (IRD) category of theories describes the interdependency of interactions between different actors in institutions, which in the long run results in permanent behavioural rules. Institutions are regarded as multifaceted, durable social structures, made up of symbolic elements, social activities and material resources. They are relatively resistant to change, and tend to be transmitted across generations, to be maintained and reproduced. At times, the normative behavioural rules are amenable to change in order to influence policy outcomes [29,30]. The outcomes can be changed by changing the rules of the game through communication, regulations, hierarchical relations, facilities (e.g. physical proximity) or actor positions.

In theories of Blurring the Boundaries, actors are given access to the internal structures and cultures of other communities, which can help them get acquainted with these other cultures, to develop a climate of trust, to mitigate the inequalities among partners, and to strengthen personal ties [26,31-34]. Wehrens et al [35,36] stressed the importance of partnership with free space to negotiate about institutional demands and to find consensus. In this respect, Warner [37] speaks of neutral “white spaces” where interorganizational barriers can be removed and trust can be built.

## Method

### Study design

An interpretative hermeneutic approach [38,39] was used to interpret the phenomenon of collaboration at the nexus. According to Gadamer, hermeneutic interpretation can help provide a deeper understanding of working at the nexus and is suitable for exploring new meaning. The interpretation is based on “lived” understanding, which means that the researcher is an interpreter from the start. The results are interpreted based on assumptions from the viewpoint of the current culture and tradition of working at the nexus [40,41]. In our case, the assumptions were that the practical strategies that have been developed have positive effects on the collaboration if they match the basic principles of the ANT, IRD and BB theories. The interpretative hermeneutic approach aims to reach some kind of “fusion of horizons”, enriching the initial understanding about collaboration at the nexus, with the interpreter’s knowledge as background [38].

### Data

Our process of interpretation used both quantitative and qualitative data, collected between May 2006 and December 2010. Quantitative data was derived from records of the outcomes of practical strategies in terms of numbers of activities and participants. Qualitative data was derived from (1) newsletter bulletins and a progress report containing unstructured interviews with ACC participants at the strategic, tactical and operational levels (n=34); (2) observations made by the researcher, which were documented in progress reports (n=3), formal (n=13) and informal documents (n=20) and (3) minutes of meetings with partners involved in the ACC at strategic level (n=34) and at tactical level (n=14). All documents together served as the data sources for the present manuscript.

### Data analysis

Manual qualitative analyses were used for the data collected in the individual interviews, the reports, the formal and informal documents and the minutes. The data were analysed by the first author using the interpretative hermeneutic circle [41,42], i.e. moving from the ACC as a whole, using basic principles of the three theories, to the individual parts, i.e. the practical strategies, and from the individual parts back to the whole. Moving back and forth between the whole and the parts leads to the formation of a common opinion. In this way, we attempted to clarify something that had been unclear so far. The findings are presented below for each system level in the form of short narratives, in which events are structured by assigning significance to the practical strategies realized, in terms of their nature and numbers. This is followed by an interpretation based on the basic principles of the three theories used: (1) ANT, i.e. the creation of a network during the stage of problematization, and the process of making and re-making engaged in by the actors in the network; (2) IRD theories, i.e. the initiation of new tasks, rules, performance indicators, facilities and norms; and (3) BB theories, i.e. contributions to a climate of trust, mutuality and dialogue. Direct quotes are shown in italics, with the sex and age of the interviewees given in brackets. The Maastricht Medical Ethics Committee has stated that no consent is required.

## Results

### Strategic level: Head in the clouds, feet firmly on the ground

The partners involved formed a coalition (the ACC) to address the challenge of crossing the boundaries of policy, practice and research and creating reciprocity. They succeeded to converge on the topic of quality improvement in local public health, which can be considered as the obligatory passage point. The formal coalition, i.e., the steering committee, met frequently, and its members participated in lobbying efforts on 24 topics (Table 1, strategy 1). The formal signing of the contractual agreement between the municipalities, the university’s Board of Governors and the Board of the PHS was marked by a kick-off meeting held in 2007, involving 65 participants from the three domains. There was a shared feeling of innovative challenge, expressed by one municipal alderman as follows: *“The ACC is the right answer to the [unfavourable] health figures in this region, and we hope it will start a dynamic process that is compatible with the ambitions of the life science campus.”* (M, 50). Expectations were high, although one alderman said: *“The ACC is intended to help the disadvantaged, not to produce PhD theses.”* (M, 50). The collaboration was not a mandate, but was seen as an opportunity for mission-driven professionals (strategies 2 and 3). Some senior academics acknowledged the added value of entering into personal contacts with the municipal authorities: *“Now it’s easier to negotiate about local public health research activities*.*”*(M, 54). Records show that in 2009, more persons (N=115) from policy, practice and research had become involved in the network symposium than in 2007 (strategy 4). The Dean of the Faculty of Health, Medicine and Life Sciences promised to continue investments, stressing that *“You can’t stop a programme of such a magnitude.”*(M, 48). Seven ambassadors, viz. one mayor and six aldermen, took up their role of promoting the different ways in which PhD research evidence can be used in the context of the policy making process. However, due to local elections and time constraints, only two of them were actually engaged in this in 2010 (strategy 5). One of the aldermen confessed that “*I still intend to collaborate but real action has faded away due to my very overloaded political agenda.”* (M, 50). In ten formal meetings of the Board of the regional PHS, senior academics were invited to facilitate conversations and dialogue about relevant health issues in its local context. These discussions were highly appreciated by the politicians (strategy 6). In order to maintain commitment at the strategic level, attention was frequently stimulated through newsletters (N=35), publications (N=201), presentations (N=24), content-related interface meetings (N=6) and website information (strategies 7-11).

### Tactical level: torn between two lovers

Managers were assumed to be at the heart of the collaborative and to be the accelerators of knowledge sharing. They were asked to create the conditions for knowledge sharing e.g., by providing time, budgets, skills training, multidisciplinary dialogue, formal and informal consultations, new projects at the nexus, advocacy for the relevance of knowledge sharing, benefits for personal careers, and a research and development team to support the leader of the ACC programme. Managers were stimulated to initiate an open-minded, actively learning and innovative organization that would be willing to go beyond routine. Each year, managers were required to draw up a plan consisting of practice- or policy-based research projects to be implemented, a number of student internships in practical settings, shared training programmes or educational support for the Bachelor’s and Master’s degree programmes, and finally, new opportunities to meet each other. However, managers tended to prioritize daily routine issues in order to meet production agreements concluded with local government, practice institute or the university Board. A professor who had to manage his department said: *“Scientists have to score, and if I want to score, I can’t do regional research, as the results then often do not arouse national or international interest.”* (M, 54). A manager of the PHS said: *“We managers are asked to allocate time to collaborative efforts and to organize regular meetings as a thematic group. We do try to do so, but we don’t really perceive this as an urgent matter, and we don’t allocate the time.”* (M, 49) Annual plans were only drawn up in 2009 (strategy 12). Nevertheless, managers agreed to allocate time to allow their staff to write new practice- or policy-based research proposals.

Many proposals were accepted (N=29 out of 37), which made the collaborative rather successful in this respect (strategy 13). Most of the material resources of the network, such as dual-staff appointments (N=17), flexible workstations (N=8), provision of e-library and software packages, access to health data files, and the provision of role models through special awards (N=3), were indeed implemented (strategies 14-16). Managers of the policy sector regularly raised questions about the ACC (N=33), as is evident from the minutes of routine meetings between practice and policy managers. These questions were merely informative, and not intended to discuss content (strategy 17). Managers endorsed the importance of knowledge sharing but found it difficult to find ways to change budgets without incurring additional expenditure. On the other hand, 13 out of 16 masterclass trainees and 10 out of 16 PhD students were supported by their manager of the practice institute (strategy 18). One manager confessed: *“If we want to engage in practice-driven research, but we can’t allocate staff to learn how to do that, we’re not getting anywhere. But as managers, we’re often in a real predicament about this”* (M, 49).

### Operational level: from hands-on action to switched-on grey matter

Selection criteria used for the recruitment of PhD researchers (N=10) emphasized previous work experience in practical or policy settings, as such characteristics may contribute to the willingness to cross borders to extend personal networks. Most candidates saw it as an opportunity to do PhD research in practice; as one of them said: *“We do want do work in a more evidence-based fashion, after all, and we need to ask ourselves how we can improve our work.”* (F, 42). And another PhD student said: *“The direct ties with practice also mean that research findings can be more rapidly translated into practical measures.”* (F, 38). The PhD students spent fifty percent of their time on research and fifty percent on their regular practical work. The same was true for the masterclass students. These are professionals from the practice or policy domain who got the opportunity to be trained in doing small-scale practical scientific research. All were eager to learn but felt insecure at the same time, as they were ignorant of the requirements of the scientific world, as expressed by one masterclass student: *“It certainly was stressful at first, especially when I was still busy collecting data and I had little time to examine aspects of science.”* (F, 49). Whereas most of them adopted a rather distant and cautious attitude towards scientific staff at first, their confidence rose over the years, and they became more self-assured in their personal contacts (strategy 19). Although some of them liked to “switch on the grey matter”, they all complied when colleagues asked for their assistance with urgent practical matters, sometimes at the request of their managers. One PhD student said: *“I know when I have to put my research on the back burner for a while. Sometimes, things like swine flu simply have to take precedence.”* (F, 38). Multidisciplinary working groups were organized to support each PhD candidate, consisting of the involved manager at the institute where the PhD candidate was appointed, the senior academic supervisor and a policy officer (strategy 20). Progress was made when senior academics realized the added value of practice-based research. One of them expressed this as *“You need eyes in practice to see what is actually going on to create innovative ideas.”* (M, 47) This resulted in research ideas that turned into successful grant proposals (N=29) (strategy 13). However, the involvement of the policy and practice domains in multidisciplinary groups could not be maintained until the end of each PhD project. Most groups ended up being left with just the PhD student and the supervisor, which is nothing more than the usual situation for PhD candidates in the scientific community. Although oral presentations at scientific conferences (N=117) were usually successful (strategy 21), the requirements of the scientific world to publish in highly cited international journals were an almost impossible endeavour for PhD students in practice (strategy 22). Sometimes academic supervisors lowered the bar somewhat by accepting submissions in Dutch peer-reviewed journals.

### Moving between all domains at all levels: many strings to the bow

The leader of the ACC programme had to move between all domains, in the horizontal as well as vertical directions (strategy 23). Contacts with managers from the policy domain were indirect, through staff members of the practice domain. Leadership and social entrepreneurship helped to put locally relevant topics on a shared public health agenda, examples being the integrated health policy to tackle overweight, school health promotion to reduce the prevalence of smoking and alcohol consumption, community health promotion to improve lifestyles, and population health statistics and forecasts to contribute to local policy development. The programme leader wrote many new grant proposals, always cooperating with a counterpart from the research domain. She said: *“Fortunately I know a lot of people at the university, and I manage to get them involved in research proposals, even of they don’t yield any immediate revenues, as you can never be sure of those. You never know whether a grant proposal will be accepted, and there’s a lot of competition.”* (F, 54). However, assistance from the practice domain lagged behind because practitioners lacked the necessary competence or the relevant manager could not allocate staff to this task. The programme leader functioned as a mediator to develop a climate of trust and to support other partners in a process requiring considerable effort over a rather long period of time. She had to play on many chess boards simultaneously.

### Interpretation in terms of theories

In terms of the Actor-Network theory, the original initiators of the ACC network were able to convince focal actors to define the obligatory passage point i.e. the need for evidence-based public health and construct a network at the time of problematization. All actors agreed that the network was worth building. Thereby, management support became indispensable. However, their involvement was non-committal and there were no sanctioning tools. After all, the network was not an obligation but rather an opportunity. Managers perceived time constraints, they were implicitly opposed to the ACC, and they were unable to define a common action program. Actors who participated in the PhD teams constructively discussed the topics of concern. Although the number of researchers and practitioners involved increased, this was not true for civil servants. Apparently, managers of the policy sector were able to connect and create nodes neither with persons outside their own organization nor with other organizational cultures, resources or communication channels. Participation was restricted to a small group of original initiators and some focal actors, viz. the programme leader, some senior academics, PhD candidates and one manager from the practice domain who had the required social entrepreneurial capability [43,44]. The programme leader could not enforce active participation because organizations acted autonomously. The expansion of the group in terms of managers from the policy and practice domains with social entrepreneurial capability was insufficient to create successful collaboration at the nexus in the long run [45].

From the perspective of theories on Institutional Re-Design, we observed that the contractual agreement was necessary to negotiate about the balance between “give and take”, both as regards the material resources and the “soft periphery”. Most material resources of the network, such as dual-staff appointments, work stations, e-library, software packages and data sharing facilities, were implemented. So far, however, the soft periphery conditions, including the creation of a culture of lifelong learning, adjustments to existing budgets and scientific impact, agenda-setting and discussions about new research proposals to obtain practice-based evidence, have only been realized within the group of original initiators and some focal actors. Practitioners and policy officers failed to respond to calls for participation in multidisciplinary groups. Participation was not mandatory, nor was it used as an indicator in annual performance interviews. Obviously, discussions did not yield enough profits in terms of useful knowledge for practice and policy. Guidance or competence training of junior group members by senior staff was not organized and proved insufficient. Currently, Dutch universities’ performance is judged by numbers of publications in journals with impact factors. The higher the number and the impact factor, the higher the budget they receive. Therefore, the pressure on universities to publish in highly cited journals is high, but this is difficult to achieve for PhD candidates in practice. Some supervisors therefore adapted their performance indicators by accepting publications in Dutch peer-reviewed journals without an impact factor. They highly valued societal impact, while still stressing the need for scientific rigour [46]. Along the way, the network emphasised the importance of societal impact and local improvements in public health implementation, gradually creating more room for practice-based research, including tacit knowledge and a compromise mix of publications in journals with and without impact factors.

The Blurring the Boundaries theories provided a structure for mutuality in the network. So-called neutral white spaces were implemented at strategic level through the steering committee, and at operational level through many different PhD teams and masterclasses. Researchers became acquainted with real-life public health problems, practitioners learned to do scientific research in practical or policy settings, and policy officers became aware of the possible contributions by researchers, even though real involvement in terms of policy officers’ participation stagnated. Local governments are no outstanding example of “learning organizations”, and proactive leadership promoting knowledge sharing is still in its infancy, which means that the absorptive capacity for new knowledge is small. In the eyes of the policy officers, the ACC was characterized by a rather high degree of abstraction, the perception being one of indistinct task issues and personal risks. The highly valued scientific impact factors of the health academics may actually have increased the gap between the policy and research domains.

## Methodological limitations

The study used a hermeneutic approach, which implies a subjective analysis. The reliability of the findings is therefore related to the researcher’s prior understanding and her interpretation of the data. The researcher, however, had substantial experience in working at the nexus of policy, practice and research [11,13,14]. The findings and interpretations were discussed on several occasions in the national programme committee (established at the request of the Ministry of Health), the steering committee of the Limburg collaborative, and the management of the PHS. In addition, the findings were evaluated against a separate evaluation study by an independent researcher [47]. The results of this evaluation study are in agreement with the findings of our interpretative hermeneutic approach.

## Discussion and conclusion

The aim of this paper was to examine the dynamics of a knowledge-sharing network, the Academic Collaborative Centre for Public Health Limburg. The ACC Limburg tried to use a more social, adaptive and self-organizing approach to build a collaborative. This approach was facilitated by a set of practical strategies to stimulate vertical and horizontal knowledge-sharing. Our interpretation of the process made use of theories that helped us explain the knowledge-sharing process. The development of the ACC thus functioned as a case study of the way policy, practice and research can become working partners on an equal footing. So far, the literature in this field has predominantly focused on traditional knowledge-translation theories. In order to change this current dominance of knowledge translation from health academics to practice and policy [48] into a balanced two-way knowledge-sharing process, the ACC linked material resources such as persons and organizational structures to immaterial issues like communication and a learning culture, wherever possible, and at all levels. Knowledge is thus being created and performed, based on context and situation specific goals, tacit knowledge and partnership [49]. This meaning of knowledge sharing corresponds with the more recent interpretation of “integrated or interactive knowledge translation”. In integrated or interactive knowledge translation, stakeholders or potential research knowledge users are engaged in the entire research process. Researchers and research users work together to shape the research process, both in terms of content (“what works”) as well as its impact (“how it works”). This approach, also known by such terms as collaborative research, action-oriented research, and co-production of knowledge, should produce research findings that are more likely be relevant to and used by the end users [50-53].

The Actor-Network Theory and theories about Institutional Re-Design and Blurring the Boundaries helped us to unravel the dynamics. ANT, IRD and BB theories are descriptive tools that facilitate understanding of the reasons for success or failure in different contexts. A systematic analysis of practical strategies enabled us to move seamlessly from “what” to “how”.

Strengths of the ACC include its ongoing process of network formation and reformation and its horizontal and vertical intraorganizational structure. Research and practice have been able to connect and the collaborative was successful in terms of the number of common project proposals that were accepted and the number of network actors involved at the strategic and operational levels. Weaknesses of the ACC collaborative mainly concern the system readiness of the policy, practice and research domains at tactical level. Whereas stakeholders at strategic level call for innovative networks, managers do not know (or do not want to know) how to join the network. They seem to consider the whole as an add-on, not as an ongoing process to improve the evidence base of public health policy and they feel restricted because they have to meet their own organizational product agreements. ACCs need strategies that are more compatible with norms and values prevailing among managers. Such strategies must be more trialable, with more observable results and with greater scope for local implementation and personal benefit, than the practical strategies applied so far. Strategies based on IRD theories might be helpful to more actively promote involvement by managers, for instance by adding performance indicators that combine the three domains, e.g. understanding the value of collaboration with external partners and scientific contributions to societal problems. Since municipal governments are responsible for local public health, their managers should be closely involved in framing public health problems and finding specific solutions [54]. In addition to complying with the maxim “if we want more evidence-based practice, we need more practice-based evidence,” ACCs should try to make policy managers aware of another maxim: “if we want more evidence-based policy, we need more policy-based evidence”. Policy-making is a social process and evidence is socially constructed. Analyzing and promoting certain policy options is a process of facilitating conversations and dialogue between different participants in the policy process [18 p.21]. Giving managers a formal position in the steering committee or an advisory board might help them intensify and continue interactions to comply with both maxims in the long term. Giving civil servants opportunities to contribute to small-scale research, improving practitioners’ capability for scientific reasoning, and increasing researchers’ competence to increase the societal relevance of their research, can be made part of their performance, monitored by their manager and guided by senior academics. Changing the rules of the game in this direction might prevent a lack of genuine interest. However, using strategies based on IRD theories to induce or force actors to engage will not suffice to solve the lagging participation of the municipal managers in the ACC. A collaborative, deliberative network of stakeholders is required, especially where the mobilization of science is critical to decision making. Convincing municipal managers to participate in the network is a challenge for the next four years, in order to establish a supportive network for evidence-based policy and policy-based evidence in public health. Evidence-based policy is a complex and long-term dynamic process of knowledge co-production in which research knowledge can be made meaningful to society. It requires social networks to improve communication between academics, policy makers, and practitioners to achieve compromises that consist of a balanced assessment of scientific and societal relevance [46].

Joining the domains of public health policy, practice and research is considered an important ingredient of current policy making. It calls for collaborative governance, with an emphasis on solving public problems or creating public value through collaboration across traditional boundaries. To improve the evidence base of current public health policy, the managers in particular have yet to create innovative rules and attractive incentives, making professionals follow their lead.

## Abbreviations

ACC: Academic collaborative centre; ANT: Actor-network theory; BB: Blurring the boundaries; IRD: Institutional re-design theories; PHS: Public health service.

## Competing interests

The authors declare that they have no competing interests.

## Authors’ contributions

MJ contributed to the design and conduct of the study, monitored data collection, helped interpret the results and drafted the manuscript. EdL, MH and NdV helped critically revise the manuscript in terms of content. All authors read and approved the final manuscript.
